# Effect of Induced Myopia on the Vestibulo-ocular Reflex Evaluated by Ocular Vestibular Evoked Myogenic Potential

**DOI:** 10.18502/jovr.v19i2.10910

**Published:** 2024-06-21

**Authors:** Mahdi Mazloom, Javad Heravian Shandiz, Sadegh Jafarzadeh, Jamshid Jamali, Hamed Momeni-Moghaddam

**Affiliations:** ^1^Department of Optometry, School of Paramedical and Rehabilitation Sciences, Mashhad University of Medical Sciences, Mashhad, Iran; ^2^Refractive Errors Research Center, Mashhad University of Medical Sciences, Mashhad, Iran; ^3^Department of Audiology, School of Paramedical and Rehabilitation Sciences, Mashhad University of Medical Sciences, Mashhad, Iran; ^4^Department of Biostatistics, School of Health, Mashhad University of Medical Sciences, Mashhad, Iran; ^5^Rehabilitation Sciences Research Center, Zahedan University of Medical Sciences, Zahedan, Iran; ^7^http://orcid.org/0000-0001-9060-1843

**Keywords:** Myopia, Ocular Vestibular Evoked Myogenic Potential, Refractive Errors, Vestibulo-ocular Reflex

## Abstract

**Purpose:**

The possible effects of refractive errors on vestibulo-ocular reflex (VOR) has been a conflicting issue. The aim of this study was to evaluate the effects of induced myopia on VOR using the ocular Vestibular Evoked Myogenic Potential (oVEMP).

**Methods:**

In this cross-sectional quasi-experimental study, 35 emmetropic and normal subjects with the mean age of 23.89 ± 3.93 (range, 20–40 years) without any ocular, nervous system, and vestibular disorders, underwent the oVEMP test in the comprehensive rehabilitation center of Mashhad University of Medical Sciences. The oVEMP was performed under five different conditions of testing binocularly, monocularly, and when myopia was induced with the use of spherical lenses of +1.00, +3.00, and +5.00 diopters, respectively. There were 2 to 5 min of rest with closed eyes after each condition to avoid adaptation, fatigue, and any other sources of bias. Mean latencies of oVEMP waves (N1 and P1) and amplitudes of N1–P1 complex were measured.

**Results:**

There was no significant difference between the right and left sides (*P* > 0.05). The induced myopia significantly increased the N1 and P1 latencies using lenses of +1.00, +3.00, and +5.00 diopters but the amplitudes of N1–P1 complex were not influenced by the different amounts of induced myopia. There was no significant difference among the different conditions of induced myopia either (*P* > 0.05).

**Conclusion:**

Induced myopia could affect the VOR due to prolonging the latencies of oVEMP waves. However, the amplitudes were not affected and the effects of multiple degrees of induced myopia were not significantly different.

##  INTRODUCTION

Thevestibulo-ocular reflex (VOR) stabilizes the gaze during head movements by holding the image steady on the fovea. The VOR causes conjugate eye movements at the same speed but in the opposite direction of head movements, thus enabling the person to perceive a clear image while moving.^[1,2]^ The VOR is made of a reflex arc which consists of three neurons from vestibular organs to vestibular nuclei and eventually to extraocular muscles.^[3]^ Any disruption in VOR could result in imbalance and oscillopsia, and blurred vision while walking or driving.^[4]^

Myopia is the most prevalent refractive error worldwide, and its incidence is increasing particularly in young adults and children.^[5,6]^ The visual system provides information about any movement of the image on the retina which plays an important role in VOR precision and is required for VOR recalibration. A decrease in the provision of this information results in diminished VOR, and imbalance.^[7,8,9]^ Uncorrected myopia which causes blurred distance vision may be one of the causes of decreased visual inputs and may therefore affect VOR. Moreover, when spectacles are worn, and objects are seen from a line of sight that is not the same as the principal axis of the lens, the prismatic effect occurs. Therefore, people who wear spectacles with minus dioptric power may need less amount of eye rotation to make up for a certain amount of head rotation in the opposite direction. These changes lead to a lower VOR gain. There is no prismatic effect and consequent change in VOR gain when contact lenses are used because they move with the eyes.^[10]^

The effects of myopia on VOR tests have been reported in previous studies.^[11,12]^ For instance, the results of the caloric test in myopic subjects who used spectacles were hypoactive and such responses were significantly more common in myopic spectacle users than in subjects with normal vision.^[12]^ Electronystagmography and rotational chair tests also showed abnormal VOR gains in myopic subjects who habitually wore spectacles.^[11]^ The results of the Romberg test of standing balance indicated that abnormal results including imbalance were significantly more common in subjects with uncorrected refractive errors than subjects with normal vision.^[13]^ All these studies demonstrate that refractive errors are associated with alterations in VOR and imbalance. The ocular Vestibular Evoked Myogenic Potential (oVEMP) is one of the common tests for VOR evaluation.^[14,15,16]^ The oVEMPs are reflexes that are dependent on the vestibular system. They are recorded from inferior oblique muscles by stimulation to the contralateral ear.^[17,18]^ There are different ways of stimulating vestibular organs such as using air-conducted sound. Muscle activities can then be recorded from surface electrodes. This is similar to the recording of visual evoked potentials where the electrode impedances must also be lower than 5kΩ.^[19]^ The oVEMP is very useful for evaluating VOR and examining patients suffering from vestibular vertigo.

The evaluation of VOR is the standard means of examining patients complaining of symptoms related to both visual and vestibular systems.^[4]^ However, less attention is paid to the refractive status of the examined patients in these tests. It is rarely noticed whether these patients have refractive errors, although few studies demonstrated the possible effect of refractive errors on the results of these tests. The aim of this study was to evaluate the effects of different degrees of induced myopia on VOR by using the oVEMP test.

##  METHODS

### Participants

Thirty-five healthy subjects including staff members of the comprehensive rehabilitation center of Mashhad University of Medical Sciences voluntarily entered this cross-sectional quasi-experimental study in 2020. They were given explicit information about the study, considering their level of education and understanding. Informed consent was obtained and the experiments adhered to the Declaration of Helsinki.

A convenience sampling was used. Inclusion criteria were candidates aged between 20 and 40 years of age, refractive error in the range of emmetropia (–0.50 < spherical equivalent < +0.05), 6/6 uncorrected visual acuity in both eyes, and the feasibility of recording the oVEMP. Exclusion criteria were candidates having any systemic or ocular disorders including cataract, glaucoma, and retinopathy, being on sedatives and CNS medications, history of ear disorders, vertigo, dizziness, imbalance, head and neck trauma and anomalies. The research ethics committee of Mashhad University of Medical Sciences approved this study (Ethics code: IR.MUMS.REC.1399.625).

### Procedure

At first, ophthalmic and vestibular histories were taken from the participants, and then they underwent screening tests such as gaze, Halmagyi head impulse and Romberg tests to evaluate the normal function of vestibular system. The refractive status was determined using an auto refractometer (Nidek AR-310A, Japan). Retinoscopy (Heine Beta 200, Germany) was performed with working distance lenses (+1.50 diopters) placed in the trial frame for accommodation control and if any latent hyperopia was realized, the refraction results were refined. Subsequently, slit-lamp examination and direct ophthalmoscopy were performed to exclude subjects with ocular diseases. The oVEMP test (Interacoustic EP25, Denmark) was used to evaluate the VOR. The active, reference, and ground disposable electrodes were placed on the upper area of forehead, on the infraorbital rim (1 cm below the pupil), and on the lower area of the forehead, respectively. The vestibular organs were stimulated with air-conducted sound by insert phones using 500Hz tone bursts. The stimuli were presented to one ear at once to record the oVEMP wave in the contralateral inferior oblique muscle. The subjects were asked to look upward, stare at a marked target on the ceiling, and keep their gaze during the measurements. This caused the belly of the inferior oblique muscle to get nearer to the skin of the subjects, which resulted in enhanced tonic activity of the muscle.^[17]^ The muscle potentials were controlled with electromyography to exclude artifacts.

The first negative trough in the oVEMP wave created in approximately 10 ms is known as N1 and there is a definite positive peak (P1) following N1 in approximately 15 ms. Each wave was repeated for recording reliable waves. The latencies of N1 and P1 waves and the amplitudes of N1–P1 complex were measured in the oVEMP evaluation and the measurements were repeated under five different conditions described as follows.

The baseline measurements were recorded with both eyes open (binocular condition). The subsequent measurements were performed monocularly by occluding the eye located at the same side of the stimulus. Then, low, moderate, and high degrees of myopia were induced by placing spherical lenses of +1.00, +3.00, and +5.00 diopters in the trial frame in the next phases of measurements, respectively. Both eyes were open under these conditions. The measurements were performed immediately after each pair of lenses were placed in the trial frame to avoid adaptation and subjects were asked to rest while closing their eyes for 2–5 min at the end of each measurement; therefore, confounding factors such as fatigue, the level of cooperation in maintaining fixation, and the upward direction of gaze were all controlled.

Given the necessity for subjects to look upward during the measurements, the trial frame was strapped to their head; therefore, it was steadied, placed properly on their faces, and aligned with their direction of gaze. It also facilitated occluding one eye during monocular measurements and caused the optical centers of the lenses to be aligned with the lines of sight of the subjects when they were placed in the trial frame for inducing myopia.

### Data Analysis

The collected data were descriptively and analytically analyzed using the SPSS software, version 27. Continuous variables were expressed as mean ± standard deviation and categorical variables were reported as frequency and percentage. The Kolmogorov–Smirnov test was used to assess the normality of the distribution of the quantitative variables. The corresponding variables in the stimulation of the right and left ears were compared using the paired sample *t*-test and the Wilcoxon test. The variables were compared between the baseline and monocular conditions using the paired sample *t*-test and the Wilcoxon test. The generalized estimating equations (GEE) model was used to compare the variables between the baseline condition and the conditions of low, moderate, and high amounts of induced myopia. A *P*-value of 5% or less was considered to be statistically significant.

**Table 1:** Comparison between two ears on the measured latencies of N1 and P1 waves and the amplitudes of N1–P1 complex under baseline, monocular, and induced myopia conditions (using lenses of +1.00, +3.00, and +5.00 diopters)


**Parameter**	**Testing condition**	**Right eye (Means ± SD)**	**Left eye (Means ± SD)**	**Total (Means ± SD)**	***P*** **-value**
Latency N1 (ms)	Baseline	12.06 ± 2.30	12.09 ± 2.20	12.07 ± 2.23	0.641
	Monocular	12.27 ± 2.39	12.36 ± 2.14	12.31 ± 2.25	0.505
	+1.00D	12.33 ± 2.35	12.30 ± 2.19	12.31 ± 2.26	0.864
	+3.00D	12.46 ± 2.36	12.05 ± 2.17	12.25 ± 2.26	0.128
	+5.00D	12.24 ± 2.37	12.38 ± 2.07	12.31 ± 2.21	0.369
	*P*-value	0.057	0.180	–	–
Latency P1 (ms)	Baseline	17.02 ± 0.83	16.89 ± 1.20	16.96 ± 1.03	0.891
	Monocular	17.13 ± 1.18	17.30 ± 0.98	17.21 ± 1.08	0.523
	+1.00D	17.24 ± 1.06	17.25 ± 1.21	17.25 ± 1.13	0.832
	+3.00D	17.19 ± 1.02	17.10 ± 1.06	17.15 ± 1.03	0.818
	+5.00D	17.20 ± 1.11	17.32 ± 0.75	17.26 ± 0.94	0.640
	*P*-value	0.017	0.305	–	–
Amplitude N1–P1 ( μ V)	Baseline	3.38 ± 2.93	3.58 ± 3.58	3.48 ± 3.25	0.833
	Monocular	3.40 ± 3.08	3.14 ± 3.43	3.27 ± 3.24	0.375
	+1.00D	3.56 ± 2.81	3.58 ± 3.61	3.57 ± 3.21	0.404
	+3.00D	3.05 ± 2.84	3.44 ± 3.24	3.25 ± 3.03	0.630
	+5.00D	3.00 ± 2.56	3.66 ± 3.70	3.33 ± 3.18	0.837
	*P*-value	0.621	0.687	–	–
	
	
ms, milliseconds; μ V, microvolt					

**Table 2:** Comparison of the measured latencies of N1 and P1 waves and the amplitudes of N1–P1 complex between the baseline and monocular conditions


**Paired samples statistics**		**Difference**			**Statistical test**
	**Means ± SD**	**Statistical test**	**Means ± SD**	**Median (interquartile range)**	**Z (** ***P*** **-value)**
N1B	12.07 ± 2.23	Wilcoxon	–0.24 ± 0.94	–0.33 (–0.34)	(0.020 ${\;}^{*}$ ) –2.322
N1M	12.31 ± 2.25		
P1B	16.96 ± 1.03	Wilcoxon	–0.26 ± 0.90	–0.33 (–0.33)	(0.008 ${\;}^{*}$ ) –2.638
P1M	17.21 ± 1.08		
ampB	3.48 ± 3.25	Paired *t*-test	0.21 ± 1.3	0.33 (0.41)	(0.178) 1.360
ampM	3.27 ± 3.24		
	
	
B, binocular; M, monocular; amp, N1–P1 amplitude ${\;}^{*}$ Significant at 0.05					

**Table 3:** Comparison of the measured latencies of N1 waves between the baseline condition and each condition of induced myopia


**(I) Condition**	**(J) Condition**	**Mean difference (I–J)**	**Std. error**	***P*** **-value**
Base	+1	–0.24	0.10	0.015 ${\;}^{*}$
	+3	–0.18	0.10	0.067
	+5	–0.24	0.10	0.015 ${\;}^{*}$
+1	+3	0.06	0.11	0.563
	+5	0.00	0.11	0.974
+3	+5	–0.06	0.11	0.617
	
	
${\;}^{*}$ Significant at 0.05				

**Table 4:** Comparison of the measured latencies of P1 waves between the baseline condition and each condition of induced myopia


**(I) Condition**	**(J) Condition**	**Mean difference (I–J)**	**Std. error**	***P*** **-value**
Base	+1	–0.29	0.13	0.023 ${\;}^{*}$
	+3	–0.19	0.11	0.078
	+5	–0.30	0.12	0.012 ${\;}^{*}$
+1	+3	0.10	0.13	0.455
	+5	–0.02	0.13	0.903
+3	+5	–0.11	0.12	0.354
	
	
${\;}^{*}$ Significant at 0.05				

##  RESULTS

Thirty-five emmetropic subjects (16 men and 19 women) with the mean age of 23.89 ± 3.93 (range, 20–40 years) participated in this study and oVEMP waves were recorded in all of them [Figure 1]. The latencies of oVEMP (N1 and P1 waves) and the amplitudes of N1–P1 complex were measured under baseline, monocular, and induced myopia conditions (using lenses of +1.00, +3.00, and +5.00 diopters). Because there was no significant difference in the data between the right and left sides (*P* > 0.05), the findings of the two sides were evaluated as a whole and 70 cases were examined. The obtained results are demonstrated in Table 1. Then, the data of the baseline and monocular conditions were compared using the paired sample *t*-test and the Wilcoxon test [Table 2].

The comparisons demonstrated that occlusion of one eye significantly prolonged the latencies of N1 and P1 waves. However, the amplitudes of N1–P1 complex were not significantly different between the baseline and monocular conditions. The results of the GEE model demonstrated that the mean N1 ( $\chi^{2}$ = 9.014; *P*-value = 0.029) and P1 ( $\chi^{2}$ = 8.080; *P*-value = 0.044) latencies were significantly different between the baseline condition and conditions of induced myopia. The results of the Fisher's least significant difference (LSD) post hoc test for assessing the changes in the mean N1 and P1 latencies following inducing low, moderate, and high amounts of myopia are illustrated in Tables 3 and 4, respectively. However, the GEE model did not show any significant differences in the mean amplitudes of the N1–P1 complex between the baseline condition and conditions of induced myopia ( $\chi^{2}$ = 3.971; *P*-value = 0.265). The results of the LSD post hoc test for assessing the changes in mean amplitudes of the N1–P1 complex following inducing different levels of myopia are illustrated in Table 5.

Thus, inducing low and high amounts of myopia significantly prolonged the latencies of N1 and P1 waves but moderate amounts of induced myopia did not change the measured latencies significantly. Different levels of induced myopia did not cause any significant changes in the measured amplitudes of the N1–P1 complex. There was no significant difference in the measured latencies and amplitudes among the conditions (+1.00, +3.00, and +5.00) of induced myopia either.

##  DISCUSSION

The current study evaluated the effect of different levels of induced myopia on VOR. The oVEMP was used to evaluate VOR as a common test.^[15]^ The results demonstrated that inducing myopia with lenses of +1 and +5 diopters significantly increased the latencies of N1 and P1 waves, although lenses of +3 diopters did not have any considerable effects on these variables. The amplitudes of the N1–P1 complex were not affected in any of the conditions of induced myopia. This study also assessed the effect of occluding one eye on the outcomes of the oVEMP test. The latencies measured under the monocular condition were significantly prolonged when they were compared with those measured under the baseline condition.

A previous study^[20]^ indicated that the vestibular and visual signals were anatomically and physiologically convergent at all levels of the axis of the central nervous system. The visual system also influences the vestibular reflexes synergistically. These influences are shown distinctly when the visual signals are unavailable. For instance, rotation in the dark results in slower compensatory eye movements and lower VOR gain than rotation in the light,^[21]^ which indicates the importance of the interactions between the visual and vestibular systems. Inducing myopia and blurring retinal images changed these interactions and caused different wave latencies in the current study. When the latencies of N1 and P1 waves were compared between the baseline condition and the condition of induced myopia using lenses of +3 diopters, the *P*-values were near to the significance level (*P* = 0.067 for N1 waves and *P* = 0.078 for P1 waves); therefore, the observed increases in the latencies with lenses of +3 diopters might have been significant if a higher number of subjects had taken part in this study.

The latencies were increased under the monocular condition. There have been studies in which disrupting binocular vision caused changes in the results of VOR evaluation. Assessing VOR after one week of patching one eye in monkeys indicated alterations in VOR only in the occluded eye.^[22]^ Moreover, monocular adaptation of VOR following inducing aniseikonia with a contact lenses/spectacles combination causing magnification in the right eye and minification in the left eye was reported in another study. The subjects wore the combination for 4 hr and VOR gain was significantly reduced only in the left eye when measured immediately after removing it and in both eyes when measured in the subsequent 30, 60, 90, and 120 min, in comparison with the baseline measurements.^[23]^ The mentioned studies confirmed our findings that the latencies were increased under the monocular condition.

Previous studies^[10][11,12][13][24][25]^ examined whether refractive errors or using different lenses had any effects on VOR. The caloric, scleral coil, electrooculography, and rotational chair tests were applied to assess VOR. One of these investigations indicated VOR adaptation in response to wearing spectacles containing lenses of +5.00 diopters. One of the subjects was emmetropic, therefore, this study also demonstrated VOR adaptation as a result of inducing –5 diopters of myopia in an emmetropic subject.^[24]^ Moreover, VOR gains measured in myopic subjects who were accustomed to using spectacles were considerably less than subjects where electrooculography and rotational chair testing were performed.^[11]^ It was also reported that myopic spectacle wearers had hypoactive or reduced responses in the caloric test compared with normal subjects.^[12]^ In another study, participants with uncorrected refractive errors had a worse balance recorded in Romberg test than those with normal vision. However, the participants were not categorized according to refractive error and the percentage of myopic participants was unknown in this study.^[13]^ On the other hand, not every conducted study indicated the effect of myopia on VOR. van Dooren et al^[25]^ evaluated the effect of the daily use of correcting spectacles on VOR gain. The video head impulse test was used and the participants were classified into three groups including subjects with no visual impairment, spectacle users, and contact lens users. The VOR gain measurements were not statistically different when the three groups were compared. They found no significant difference in the VOR gain measurements between the binocular and monocular conditions either. However, the current study demonstrated the effects of induced myopia and the monocular–binocular differences. The discrepancies between the results of the current study and the one conducted by van Dooren et al^[25]^ are not necessarily caused by using different devices for evaluating VOR but may be associated with visual adaptation and central compensations. All subjects in the current study were emmetropic and the oVEMP wave was immediately recorded after myopia was induced to avoid adapting to the applied lenses, but the subjects in the van Dooren et al's study^[25]^ had been wearing spectacles to correct their refractive errors for a long time (4 months to 60 years). Therefore, they had experienced decreased visual inputs due to uncorrected refractive errors for longer periods. Central compensation mechanisms can offset the reduction in visual inputs in this extended period. van Dooren et al^[25]^ did not report the percentage of myopic subjects either. The VOR gain was evaluated only in the right eye due to the use of video Head Impulse Test (vHIT), while the left eye was covered due to possible differences in refractive errors of the two eyes. However, in the current study, both eyes were open under the conditions of induced myopia. Myopia was also induced in both eyes equally and simultaneously.

This study had the advantage of using the oVEMP test instead of other VOR tests with movement. Convex lenses were used to induce different degrees of myopia which can also cause prismatic effects, and consequently make changes in VOR. Therefore, a certain degree of head rotation causes a higher degree of eye rotation.^[10]^ However, during the oVEMP test, the vestibular organs were stimulated by sound which ensures that the heads and eyes of the subjects were stationary and did not move.

The trial frame was placed in front of the eyes in a way that the subjects saw the target only through the optical centers of the lenses and considering there was no eye or head movement during the test, prismatic effects did not occur and VOR was not affected by them. Hence the effect of myopic defocus was assessed solely.

The current study demonstrates the significant effects of induced myopia on VOR. However, the degree of the produced effects on results of the oVEMP test is clinically mild. The results of the oVEMP test in investigating vestibular lesions are mainly determined by comparing the findings of the two sides and the differences in amplitudes are primarily used. Given that induced myopia does not affect wave amplitudes and the effects of multiple degrees of induced myopia are not significantly different, there is no need to modify the evaluation protocols and no corrective factors are required.

The current study has the limitation that induced myopia creates a different physiological condition than real myopia, which may influence the results. However, inducing myopia in emmetropic subjects instead of using real myopic subjects provides the ability to control the testing conditions better and the variances between the different degrees of myopia can be compared more appropriately.

The results of this study indicate that the presence of myopia can cause significant differences in VOR results. The differences were noticed in the measured wave latencies but induced myopia did not influence the measured wave amplitudes. The effects of multiple levels of myopia were not substantially different, either. No corrective factor is suggested when myopic patients undergo the oVEMP test.

##  Financial Support and Sponsorship

None.

##  Conflicts of Interest

None.
